# Supplementary material for: An integrated multiple driver mesocosm experiment reveals the effect of global change on planktonic food web structure
Source: Commun Biol. 2022 Mar 1;5:179. doi: 10.1038/s42003-022-03105-5 (PMC8888609; doi:10.1038/s42003-022-03105-5)
Supplement: Supplementary file 2 — Supplementary Information [file 42003_2022_3105_MOESM2_ESM.pdf]

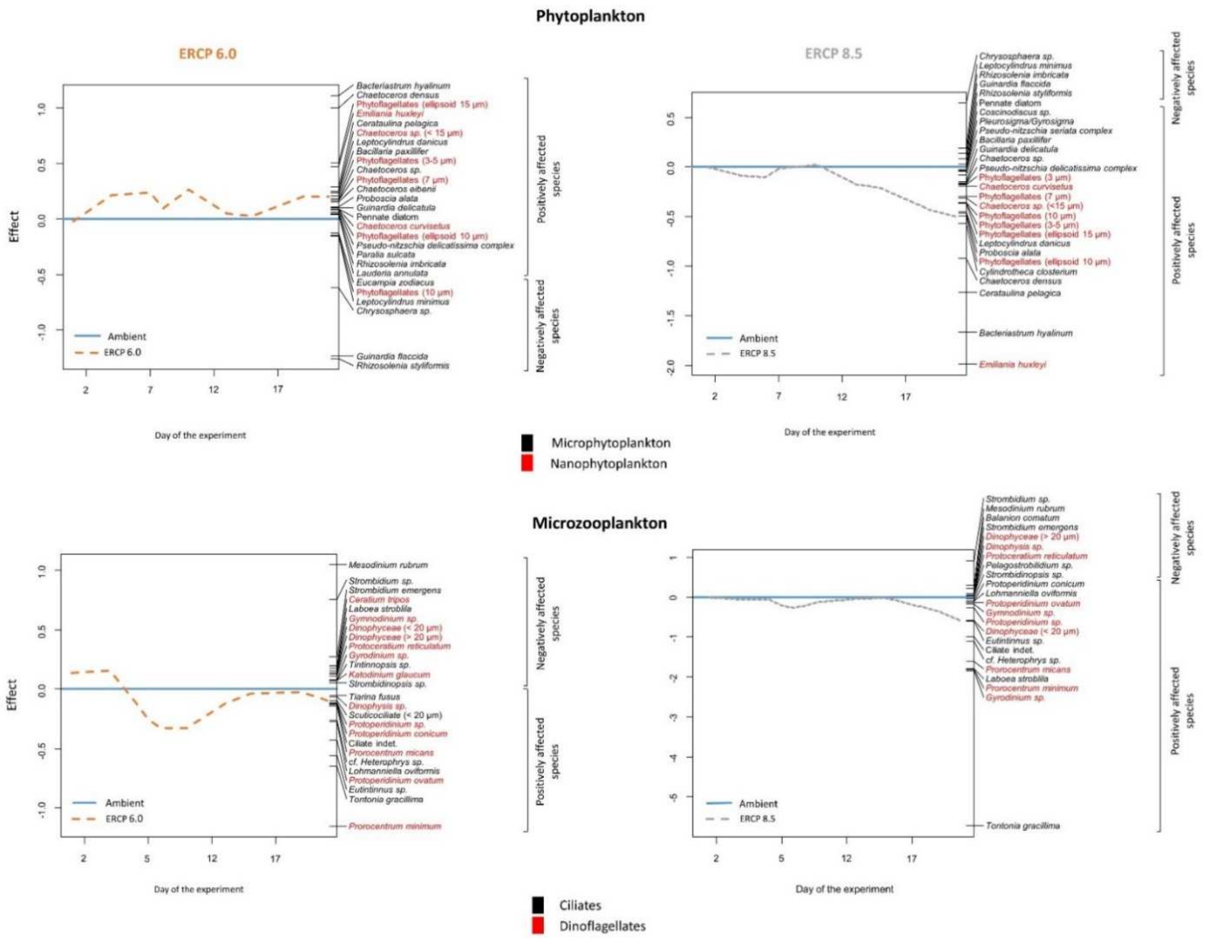

Supplementary Figure 1: Principal Response Curve of the plankton community. Graphic representation of the phytoplankton (top) and microzooplankton (bottom) community response over time in the extended Representative Concentration Pathway (ERCP) scenarios 6.0 and 8.5 in comparison to the Ambient treatment. Time and scenario explained 85% and 79% of the variation in phytoplankton community composition in the scenario ERCP 6.0 and ERCP 8.5, respectively. For microzooplankton community composition, it was 74% and 56%. For clarity, only the most affected taxa are displayed on the diagram.

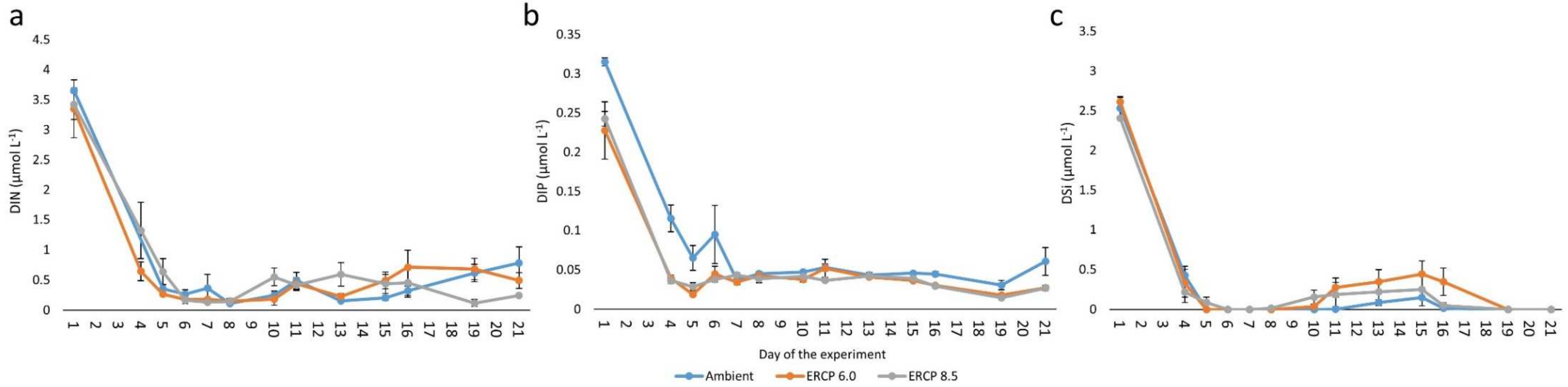

Supplementary Figure 2: Dissolved inorganic nutrient concentrations in the mesocosms during the experiment. (a) Dissolved inorganic nitrogen (DIN) = $NO_x + NH_4^+$. (b) Dissolved inorganic phosphorus (DIP) = PO_4^{3-}. (c) Dissolved inorganic silicate (DSi) = SiO_3^-; x-axis represents the days of the experiment, different colours represent the Ambient treatment and Extended Representative Concentration Pathway (ERCP) scenarios (blue = Ambient, orange = ERCP 6.0, grey = ERCP 8.5), mean ± standard deviation.

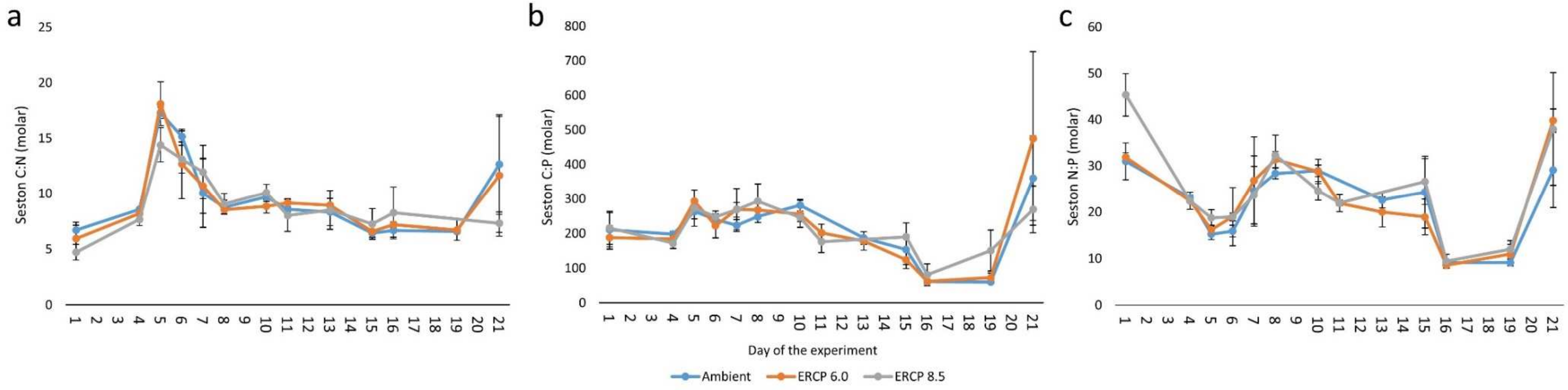

Supplementary Figure 3: Seston elemental stoichiometry. (a) Seston C:N stoichiometry (Carbon:Nitrogen). (b) Seston C:P stoichiometry (Carbon:Phosphorus). (c) Seston N:P stoichiometry (Nitrogen:Phosphorus), x-axis represents the day of the experiment, different colours represent the ambient and extended Representative Concentration Pathway (ERCP) scenarios (blue = Ambient, orange = ERCP 6.0, grey = ERCP 8.5), mean ± standard deviation.

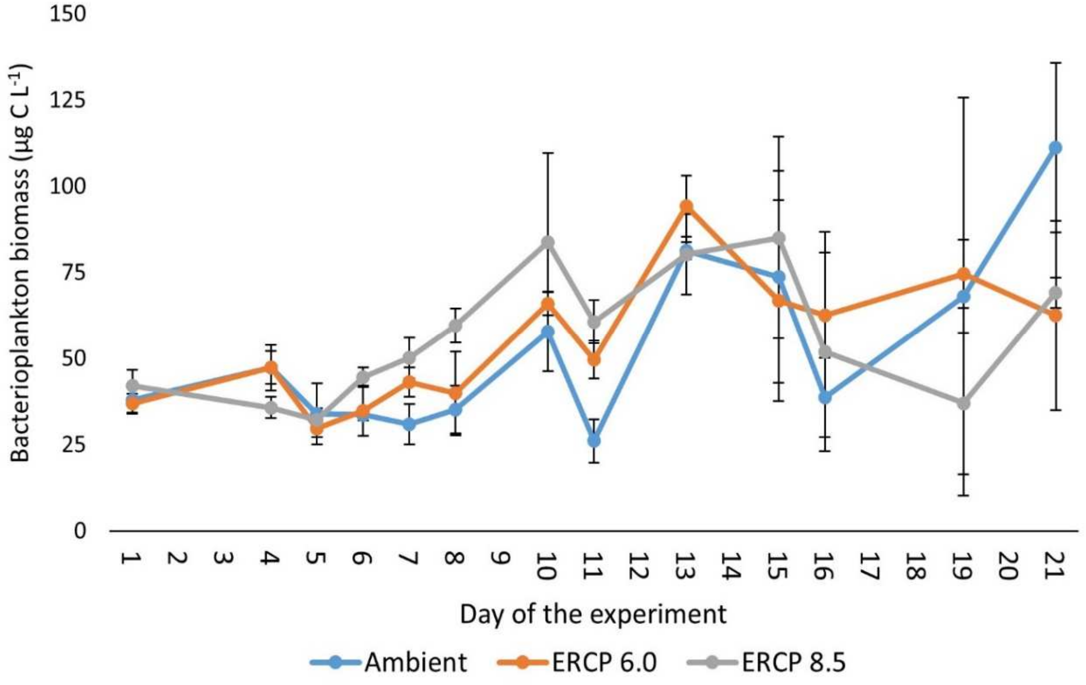

Supplementary Figure 4: Bacterioplankton biomass. X-axis represents the days of the experiment, different colours represent the Ambient treatment and Extended Representative Concentration Pathway (ERCP) scenarios (blue = Ambient, orange = ERCP 6.0, grey = ERCP 8.5), mean ± standard deviation.

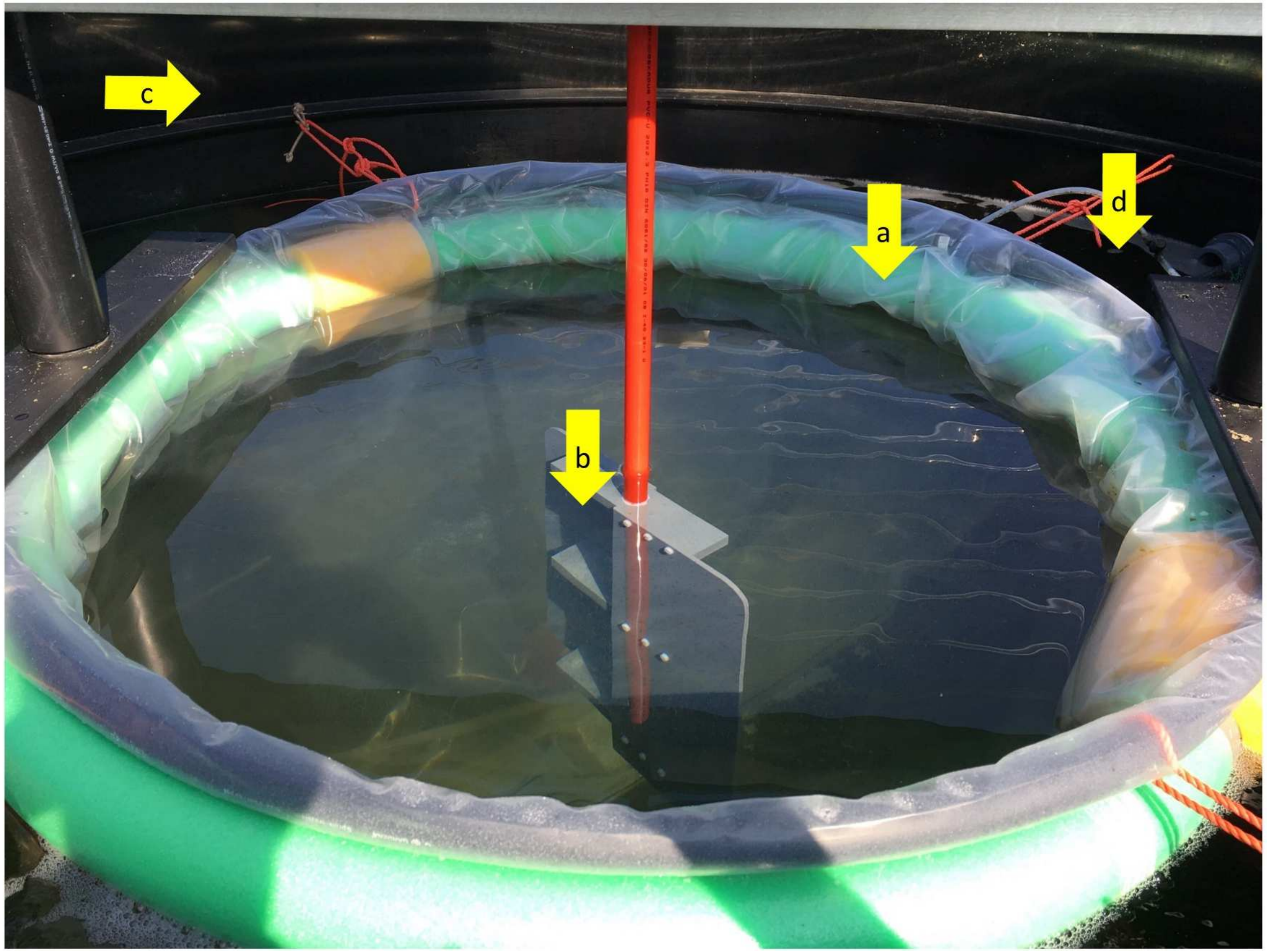

Supplementary Figure 5: Photo of the experimental setup. (a) The low-density polyethylene bag filled with seawater containing natural planktonic community, (b) The high-density polyethylene paddle for stirring, (c) the mesocosm tank and (d) surrounding temperature-controlled water bubbled with *p*CO_2-controlled air.

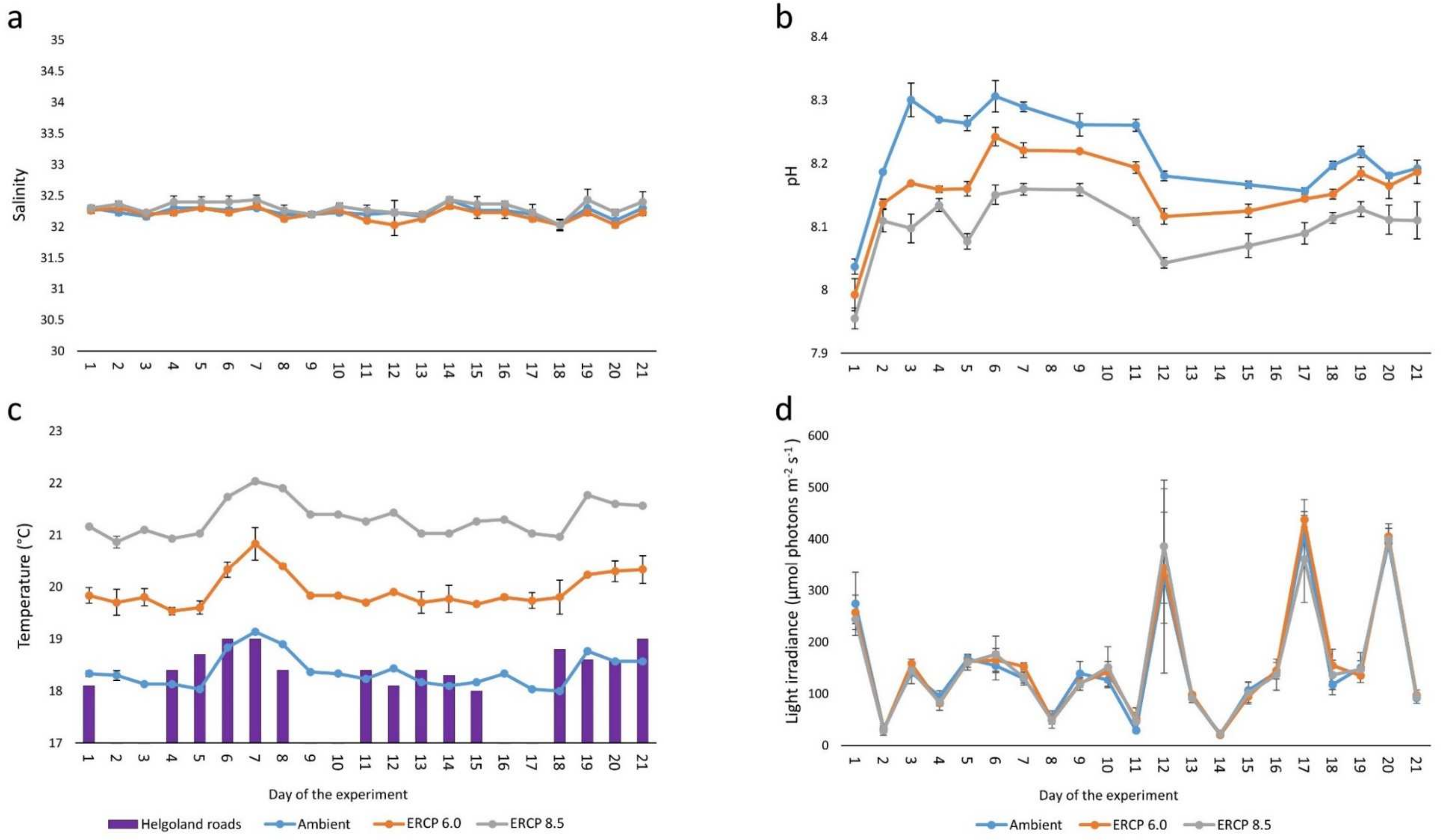

Supplementary Figure 6: Environmental conditions in the mesocosms during the experiment. (a) Salinity. (b) pH. (c) Temperature. (d) Light irradiance. Purple bars represent surface seawater temperature at Helgoland Roads; x-axis represents the days of the experiment, different colours represent the Ambient treatment and Extended Representative Concentration Pathway (ERCP) scenarios (blue = Ambient, orange = ERCP 6.0, grey = ERCP 8.5), mean ± standard deviation.
