# Supplementary material for: An integrated multiple driver mesocosm experiment reveals the effect of global change on planktonic food web structure
Source: Commun Biol. 2022 Mar 1;5:179. doi: 10.1038/s42003-022-03105-5 (PMC8888609; doi:10.1038/s42003-022-03105-5)
Supplement: Supplementary file 3 — Description of Additional Supplementary Files [file 42003_2022_3105_MOESM3_ESM.pdf]

Description of Additional Supplementary Files

File name: Supplementary Data 1

Description: *Carbonate chemistry in the mesocosms during the experiment.* Dissolved inorganic carbon (DIC), partial pressure of CO_2 (pCO_2), Bicarbonate (HCO_3^-), Carbonate (CO_3^{2-}), Carbon dioxide (CO_2) and calcite saturation state ($W_{calcite}$) were calculated based on pH and total alkalinity (TA) using CO2Sys (Pierrot et al. 2006); mean ± standard deviation.
