# Supplementary material for: An integrated multiple driver mesocosm experiment reveals the effect of global change on planktonic food web structure
Source: Commun Biol. 2022 Mar 1;5:179. doi: 10.1038/s42003-022-03105-5 (PMC8888609; doi:10.1038/s42003-022-03105-5)
Supplement: Supplementary file 5 — Reporting Summary [file 42003_2022_3105_MOESM5_ESM.pdf]

nature portfolio

Corresponding author(s): Hugo Duarte Moreno

Last updated by author(s): Dec 21, 2021

Reporting Summary

Nature Portfolio wishes to improve the reproducibility of the work that we publish. This form provides structure for consistency and transparency in reporting. For further information on Nature Portfolio policies, see our Editorial Policies and the Editorial Policy Checklist.

Statistics

For all statistical analyses, confirm that the following items are present in the figure legend, table legend, main text, or Methods section.

n/a	Confirmed	
☐	☒	The exact sample size (n) for each experimental group/condition, given as a discrete number and unit of measurement
☐	☒	A statement on whether measurements were taken from distinct samples or whether the same sample was measured repeatedly
☐	☒	The statistical test(s) used AND whether they are one- or two-sided<br>*Only common tests should be described solely by name; describe more complex techniques in the Methods section.*
☐	☒	A description of all covariates tested
☐	☒	A description of any assumptions or corrections, such as tests of normality and adjustment for multiple comparisons
☐	☒	A full description of the statistical parameters including central tendency (e.g. means) or other basic estimates (e.g. regression coefficient) AND variation (e.g. standard deviation) or associated estimates of uncertainty (e.g. confidence intervals)
☐	☒	For null hypothesis testing, the test statistic (e.g. F, t, r) with confidence intervals, effect sizes, degrees of freedom and P value noted<br>*Give P values as exact values whenever suitable.*
☒	☐	For Bayesian analysis, information on the choice of priors and Markov chain Monte Carlo settings
☒	☐	For hierarchical and complex designs, identification of the appropriate level for tests and full reporting of outcomes
☒	☐	Estimates of effect sizes (e.g. Cohen's d, Pearson's r), indicating how they were calculated

Our web collection on statistics for biologists contains articles on many of the points above.

Software and code

Policy information about availability of computer code

Data collection	*Provide a description of all commercial, open source and custom code used to collect the data in this study, specifying the version used OR state that no software was used.*
Data analysis	R software (version 3.4.3) used and codes used: glm (generalized linear model), anova (test = likelihood ratio test) and PRC (principal response curve).

For manuscripts utilizing custom algorithms or software that are central to the research but not yet described in published literature, software must be made available to editors and reviewers. We strongly encourage code deposition in a community repository (e.g. GitHub). See the Nature Portfolio guidelines for submitting code & software for further information.

Data

Policy information about availability of data

All manuscripts must include a data availability statement. This statement should provide the following information, where applicable:

- Accession codes, unique identifiers, or web links for publicly available datasets
- A description of any restrictions on data availability
- For clinical datasets or third party data, please ensure that the statement adheres to our policy

The data will be available through the PANGAEA platform. We are currently working on uploading the data and as soon as it is available we provide the access.

Field-specific reporting

Please select the one below that is the best fit for your research. If you are not sure, read the appropriate sections before making your selection.

☐ Life sciences ☐ Behavioural & social sciences ☒ Ecological, evolutionary & environmental sciences

For a reference copy of the document with all sections, see nature.com/documents/nr-reporting-summary-flat.pdf

Ecological, evolutionary & environmental sciences study design

All studies must disclose on these points even when the disclosure is negative.

Study description	We applied an integrated multiple driver design to assess the potential impact of global change on natural coastal plankton communities. We tested the influence of two future scenarios against current environmental conditions: the Ambient condition (ambient temperature and pH) and the Representative Concentration Pathway (RCP 6.0, +1.5C, -0.2 pH) and RCP 8.5 (+3.0°C, -0.3 pH), proposed by the IPCC for 2100. Additionally, as nutrient inputs are also predicted to change towards considerably higher nitrogen to phosphorus (N:P) in coastal seas, we extended the RCP scenarios (ERCP) to simulate changing nutrients regimes, with a N:P ratio (molar) of 16 (Redfield ratio) for the Ambient scenario and 25 for the both future scenarios (ERCP 6.0 and 8.5).
Research sample	The research samples was a volume of water containing natural plankton communities, which we collected in the North Sea and divided into different mesocosms to conduct our study.
Sampling strategy	We used four replicates per scenario to maximize the statistical power and enable a feasible experiment. This number of replicates is typical for the type of study we conducted.
Data collection	Physical-chemical parameters and chlorophyll a were measured daily by one of the co-authors at 9:00 AM. The other samples were always collected by the same researchers, also at 9:00 AM.
Timing and spatial scale	The experiment ran for 3 weeks and sampling took place in 1-3 days interval according to the development of the phytoplankton bloom. Sampling was more frequent when stronger changes were detected in chlorophyll a concentrations. The experiment was stopped after 3 weeks due to the end of the phytoplankton bloom in two of the three scenarios and to prevent any bottle effect.
Data exclusions	One replicate was excluded from each scenario due to damage to the mesocosm bags, which led to contamination by the surrounding water. As we initially included four replicates per scenario in our study, this loss did not significantly alter the quality of our study as the remaining three replicates still provided suficient statistical power to identify statistically differences across scenarios.
Reproducibility	All sampling and analytical methods used are standard in plankton ecology. This ensure the reproducibility of our study.
Randomization	The position of all scenario replicates was randomized to prevent confounding factors.
Blinding	All samples were identified with individual numbers and not with scenario names to ensure that all analyses were conducted blindly.

Did the study involve field work? ☒ Yes ☐ No

Field work, collection and transport

Field conditions	Seawater temperature 19.5°C, low wind speed and waves smaller than 1m.
Location	North Sea (55°01'20.0"N 7°38'41.0"E)
Access & import/export	Eight hundred litres of seawater were collected with the research vessel Uthörn and the water was transported to the AWI station on Sylt. No special permit needed.
Disturbance	No disturbances caused by the study.

Reporting for specific materials, systems and methods

We require information from authors about some types of materials, experimental systems and methods used in many studies. Here, indicate whether each material, system or method listed is relevant to your study. If you are not sure if a list item applies to your research, read the appropriate section before selecting a response.

Materials & experimental systems

n/a	Involved in the study
☒	☐ Antibodies
☒	☐ Eukaryotic cell lines
☒	☐ Palaeontology and archaeology
☒	☐ Animals and other organisms
☒	☐ Human research participants
☒	☐ Clinical data
☒	☐ Dual use research of concern

Methods

n/a	Involved in the study
☒	☐ ChIP-seq
☒	☐ Flow cytometry
☒	☐ MRI-based neuroimaging
